# Hybrid Monoterpenoid Indole Alkaloids Obtained as Artifacts from *Rauvolfia tetraphylla*

**DOI:** 10.1007/s13659-015-0074-2

**Published:** 2015-09-29

**Authors:** Yuan Gao, Dong-Sheng Zhou, Ping Hai, Yan Li, Fei Wang

**Affiliations:** BioBioPha Co., Ltd., Kunming, 650201 People’s Republic of China; Department of Chemical Engineering, Yibin University, Yibin, 644000 People’s Republic of China; State Key Laboratory of Phytochemistry and Plant Resources in West China, Kunming Institute of Botany, Chinese Academy of Sciences, Kunming, 650201 People’s Republic of China

**Keywords:** *Rauvolfia tetraphylla*, Monoterpenoid indole alkaloid, Rauvotetraphylline

## Abstract

**Abstract:**

Five new hybrid monoterpenoid indole alkaloids bearing an unusual 2,2-dimethyl-4-oxopiperidin-6-yl moiety, namely rauvotetraphyllines F–H (**1**, **3**, **4**), 17-*epi*-rauvotetraphylline F (**2**) and 21-*epi*-rauvotetraphylline H (**5**), were isolated from the aerial parts of *Rauvolfia tetraphylla*. Their structures were established by extensive spectroscopic analysis. The new alkaloids were evaluated for their cytotoxicity in vitro against five human cancer cell lines.

**Graphical Abstract:**

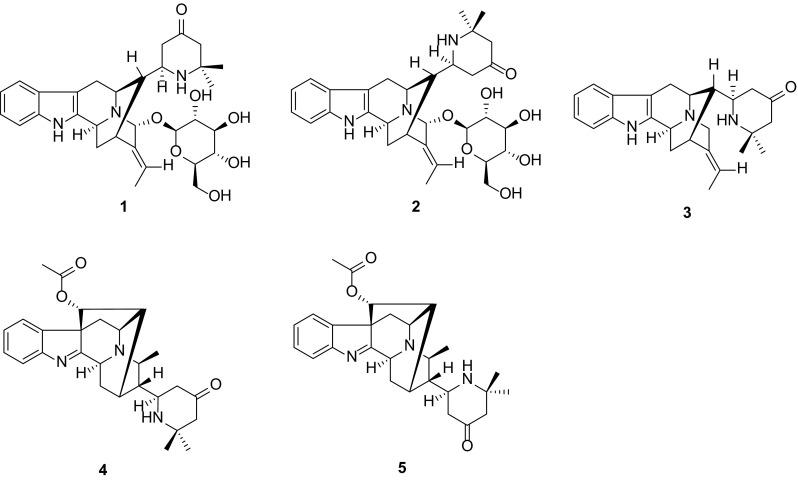

**Electronic supplementary material:**

The online version of this article (doi:10.1007/s13659-015-0074-2) contains supplementary material, which is available to authorized users.

## Introduction

*Rauvolfia* genus of the Apocynaceae family, comprising about 60 species, is mainly distributed in America, Africa, Asia, and Oceania [1]. Plants of this genus are a rich source of monoterpenoid indole alkaloids, which have attracted great interests from biological and therapeutic aspects [2–5]. As part of a BioBioPha [http://www.chemlib.cn] objective to assemble a large-scale natural product library valuable in the discovery of new drug leads from nature, previous chemical study on the ethanolic extract of *Rauvolfia tetraphylla* had resulted in the isolation of five new indole alkaloids, rauvotetraphyllines A–E [6]. Further investigation of the remaining components led to the isolation of another five new alkaloids bearing an unusual 2,2-dimethyl-4-oxopiperidin-6-yl moiety, rauvotetraphyllines F–H (**1**, **3**, **4**), 17-*epi*-rauvotetraphylline F (**2**) and 21-*epi*-rauvotetraphylline H (**5**). The present paper describes the isolation, structure elucidation, and cytotoxic evaluation of the new compounds.

## Results and Discussion

**Fig. 1:**
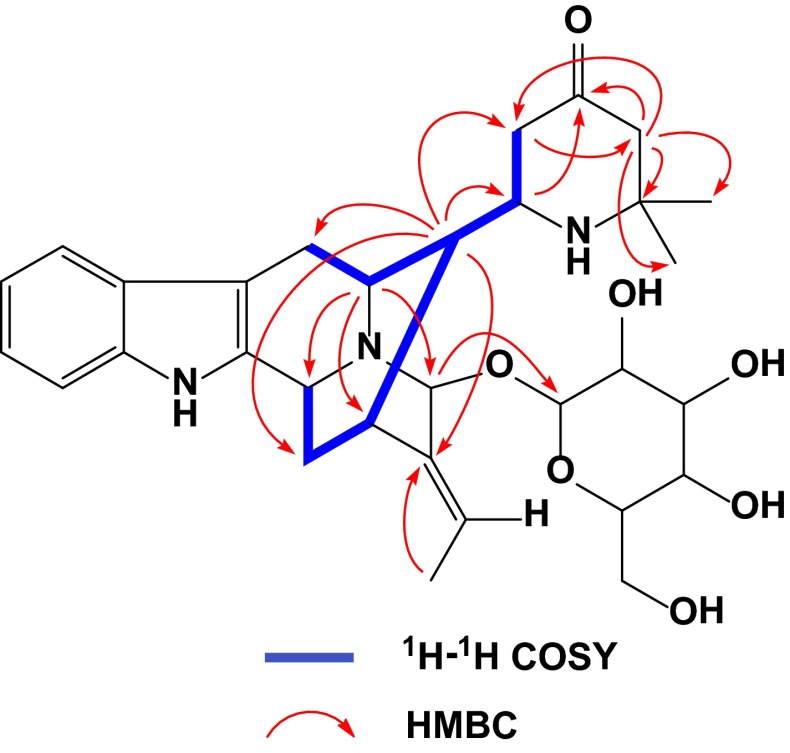
Key HMBC and ^1^H–^1^H COSY correlations of **1**

Compound **1**, obtained as amorphous powder, possessed a molecular formula of C_31_H_41_N_3_O_7_, as evidenced by HR-ESI-MS (pos.) at *m*/*z* 568.3025 (calcd for C_31_H_42_N_3_O_7_, 568.3022), in combination with NMR spectra (Tables 1 and 3), requiring 13 degrees of unsaturation. In the UV spectrum, two characteristic maxima at 225 and 281 were detected, suggesting the existence of an unsubstituted indole chromophore [7]. The IR spectrum showed the presence of OH/NH (3404 cm^−1^) functionalities. The 1D-NMR spectra (Tables 1 and 3) revealed the presence of an unsubstituted indole moiety [*δ*_H_ 7.38 (1H, d, *J* = 7.8 Hz), 7.26 (1H, d, *J* = 8.0 Hz), 7.02 (1H, dd, *J* = 8.0, 7.3 Hz), and 6.95 (1H, dd, *J* = 7.8, 7.3, Hz); *δ*_C_ 139.7 (s), 138.2 (s), 128.9 (s), 121.9 (d), 119.7 (d), 118.5 (d), 111.9 (d), and 104.0 (s)], an ethylidene group [*δ*_H_ 1.75 (3H, d, *J* = 6.9 Hz) and 5.93 (1H, q, *J* = 6.9 Hz); *δ*_C_ 14.3 (q) and 125.2 (d)], and a glucose unit [*δ*_H_ 4.64 (1H, d, *J* = 7.9 Hz), 3.29 (1H, m), 3.37 (1H, m), 3.28 (2H, m), 3.60 (1H, dd, *J* = 11.9, 4.9 Hz), and 3.74 (1H, dd, *J* = 11.9, 1.6 Hz); *δ*_C_ 103.2 (d), 78.0 (d), 77.9 (d), 75.4 (d), 71.4 (d), and 62.7 (t)]. Comparison of its ^13^C NMR data with those of rauvotetraphylline B [6] revealed a remarkable resemblance except for a prominent difference as follows: the carbon signals assigned to 4,6-dimethylpyrid-2-yl unit in rauvotetraphylline B were not present, and there was a set of newly arisen resonances [*δ*_C_ 56.5 (d), 47.4 (t), 213.2 (s), 55.7 (t), 55.5 (s), 25.6 (q), and 31.9 (q)] determined as a 2,2-dimethyl-4-oxopiperidin-6-yl moiety by HMBC correlations (Fig. 1) from H-17 to C-23, H-22 to C-23 and C-24, and H-24 to C-22, C-23, C-25, C-26, and C-27. The piperidinyl moiety was linked to C-16 through C-16–C-17 bond by HMBC correlations from H-16 to C-17 and C-22 and ^1^H-^1^H COSY correlation of H-16/H-17 (Fig. 1).

**Table 1:** ^1^H NMR Data for Compounds **1**–**3** (*δ* in ppm, *J* in Hz)

No.	**1** ^a^	**2** ^a^	**3** ^b^
3	4.67 (dd, 10.1, 2.1)	4.70 (br d, 10.5)	4.07 (br d, 10.4)
5	2.88 (dd, 7.0, 5.3)	3.20 (dd, 7.0, 5.5)	2.92 (dd, 7.5, 5.3)
6*α*	3.10 (dd, 15.2, 5.3)	3.16 (dd, 15.4, 5.5)	3.08 (dd, 15.3, 5.3)
6*β*	2.64 (d, 15.2)	2.91 (d, 15.4)	2.53 (d, 15.3)
9	7.38 (d, 7.8)	7.43 (d, 7.7)	7.44 (d, 7.6)
10	6.95 (dd, 7.8, 7.3)	6.97 (dd, 7.7, 7.2)	7.09 (dd, 7.6, 7.3)
11	7.02 (dd, 8.0, 7.3)	7.04 (dd, 8.0, 7.2)	7.13 (dd, 7.8, 7.3)
12	7.26 (d, 8.0)	7.27 (d, 8.0)	7.26 (d, 7.8)
14*α* 14*β*	2.10 (m) 1.64 (m)	2.14 (m) 1.66 (m)	2.01 (m) 1.68 (m)
15	3.26 (br s)	2.79 (br s)	3.12 (br s)
16	1.62 (ddd, 9.0, 7.0, 0.9)	1.65 (m)	1.53 (ddd, 9.4, 7.3, 0.9)
17	2.80 (ddd, 12.1, 9.0, 2.7)	2.75 (ddd, 12.2, 9.2, 2.8)	2.95 (ddd, 11.6, 9.4, 2.7)
18	1.75 (d, 6.9)	1.71 (d, 6.9)	1.65 (d, 6.8)
19	5.93 (q, 6.9)	5.95 (q, 6.9)	5.34 (q, 6.8)
21	4.96 (s)	5.03 (s)	3.55 (2H, s)
22*α* 22*β*	2.41 (br d, 12.3) 2.12 (dd, 12.3, 12.1)	2.43 (br d, 12.5) 1.95 (dd, 12.5, 12.2)	2.47 (br d, 12.7) 1.92 (dd, 12.7, 11.6)
24*α*	2.15 (dd, 13.0, 1.3)	2.16 (dd, 13.3, 0.8)	2.25 (dd, 13.1, 1.3)
24*β*	2.26 (d, 13.0)	2.29 (d, 13.3)	2.12 (d, 13.1)
26	0.95 (s)	0.98 (s)	0.99 (s)
27	1.19 (s)	1.29 (s)	1.19 (s)
1′	4.64 (d, 7.9)	4.65 (d, 7.8)	
2′	3.29 (m)	3.30 (m)	
3′	3.37 (m)	3.37 (m)	
4′	3.28 (m, overlap)	3.29 (m, overlap)	
5′	3.28 (m, overlap)	3.29 (m, overlap)	
6′a	3.60 (dd, 11.9, 4.9)	3.60 (dd, 11.9, 4.9)	
6′b	3.74 (dd, 11.9, 1.6)	3.76 (br d, 11.9)	
1-NH			8.08 (s)

^a^Measured in methanol-*d*_4_ (3.30 ppm)

^b^Measured in CDCl_3_ (7.26 ppm)

**Fig. 2:**
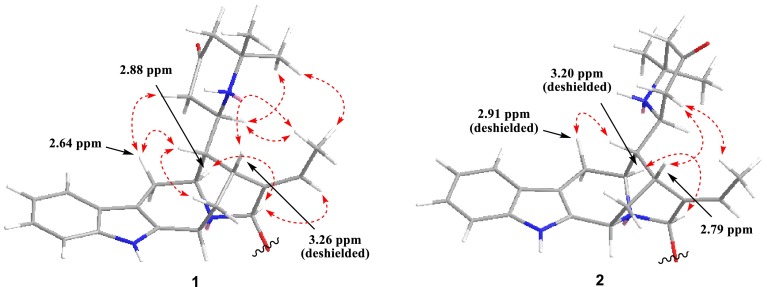
Key ROESY correlations of **1** and **2**

The relative configuration of **1** was established by NMR analysis based on computer-generated 3D drawing with minimized energy by MM2 calculation (Fig. 2). ROESY correlations of H-16↔H-6*β*/H-14*β* and H-5↔H-21 suggested that **1** had the same stereochemistry as rauvotetraphylline B. The *E*-geometry of the ethylidene was indicated from ROESY correlations of H-15↔Me-18 and H-19↔H-21. The *anti* relationship of H-16 and H-17 was suggested by the large coupling constant (*J*_16,17_ = 9.0 Hz), which could also be explained by that the molecule favors the conformation in which larger substituents are in the *anti* position. This was further supported by ROESY correlations of H-17↔Me-18. The R* configuration of C-17 was implied by ROESY correlations of H-6*β*↔H-22*β* and Me-18↔Me-26 (Fig. 2). Another noteworthy observation is that the chemical shift of H-15 (*δ*_H_ 3.26) in **1** was relatively deshielded compared to that of H-15 (*δ*_H_ 2.79) in its 17-epimer **2** (vide infra). This is attributed to paramagnetic deshielding caused by the proximity of the NH nitrogen atom to H-15 (Fig. 2). Thus, the structure of **1** was established as shown and named rauvotetraphylline F.

Compound **2**, isolated as amorphous powder, had the same molecular formula as **1** based on HR-ESI-MS (pos.), showing a quasi-molecular ion peak at *m*/*z* 568.3031 (calcd for C_31_H_42_N_3_O_7_, 568.3022). The ^1^H and ^13^C NMR spectra of **2** (Tables 1 and 3) were very similar in all respects to those of **2** except for the chemical shifts of H-5, H-6*β*, and H-15 in the ^1^H NMR spectrum. This discrepancy proved that compound **2** is a C-17 epimer of **1** while applying the same analysis carried out for **1**. The paramagnetic deshielding experienced by H-15 in **1** was now experienced by H-5 and H-6*β* instead in **2** (Fig. 2), implying the S* configuration of C-17. This was further verified by strong ROESY correlations between H-22*α* and H-15/Me-18 and no correlation between Me-26 and Me-18. Therefore, the structure of **1** was elucidated as shown and named 17-*epi*-rauvotetraphylline F.

Compound **3** was obtained as amorphous powder. Its HR-ESI-MS revealed an [M + H]^+^ peak at *m*/*z* 390.2538 (calcd for C_25_H_32_N_3_O, 390.2545), suggesting the molecular formula C_25_H_31_N_3_O. The NMR data (Tables 1 and 3) were closely related to those of **1** except for the signals of a methylene group in **3** instead of an oxygenated methine group in **1**, and the absence of a series of glucose resonances. The configuration of C-17 was designated as R* based on Me-18 showing ROESY correlation to Me-26, but no correlation to H-22. Consequently, the structure of **3** was determined and named rauvotetraphylline G.

**Fig. 3:**
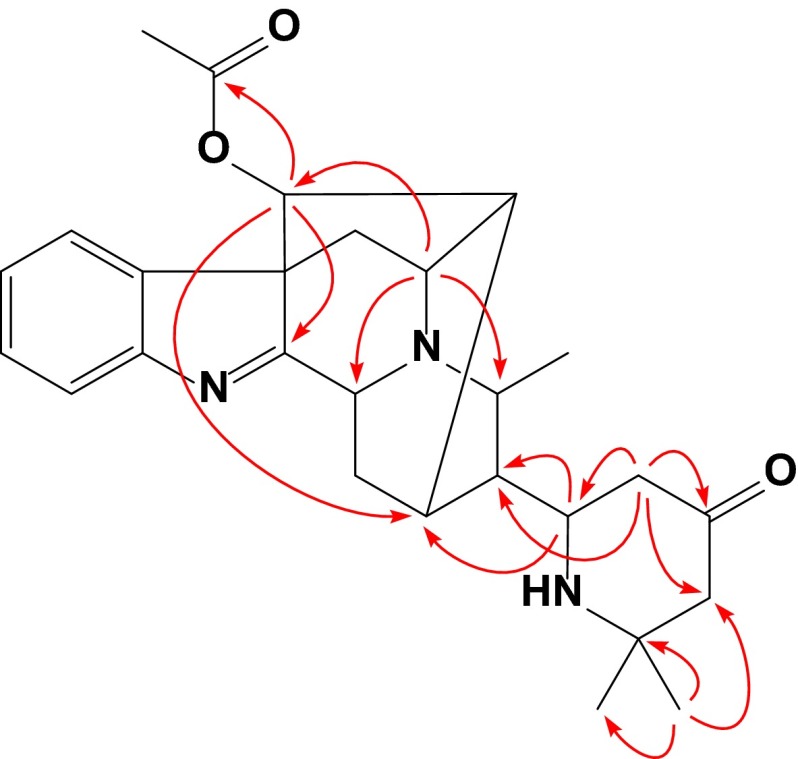
Key HMBC correlations of **4**

**Fig. 4:**
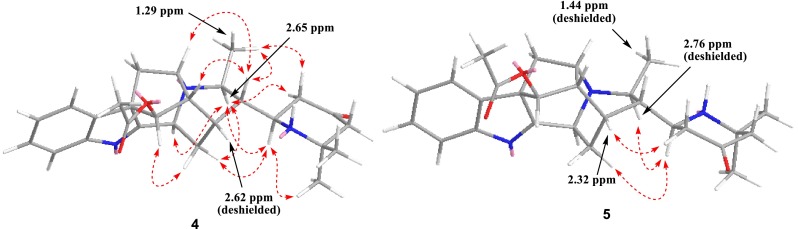
Key ROESY correlations of **4** and **5**

Compound **4** was isolated as amorphous powder. Its molecular formula was determined as C_27_H_33_N_3_O_3_ by positive HR-ESI-MS at *m*/*z* 448.2613 (calcd for C_27_H_34_N_3_O_3_, 448.2600). The ^13^C NMR data (Table 3) were very similar to those of perakine [8]. The prominent difference between them was the aldehyde group in perakine changing into a 2,2-dimethyl-4-oxopiperidin-6-yl moiety [*δ*_C_ 54.8 (d), 47.2 (t), 210.4 (s), 55.4 (t), 54.5 (s), 25.2 (q), 32.2 (q)] on the basis of HMBC correlations (Fig. 3) from H-21 to C-15 and C-20, H-22 to C-20, C-21, C-23, and C-24, and Me-26 to C-24, C-25, and C-27. The ROESY correlations (Fig. 4) of H-19↔H-3/H-14*α*, H-14*β*↔H-17, Me-18↔H-20, and H-20↔H-5/H-16 indicated that **4** possessed the same stereochemical characteristics as perakine. The R* configuration of C-21 was indicated by ROESY correlations of H-21↔H-14*α*/H-19, H-22*α*↔H-19, and H-22*β*↔Me-18, which was further supported by comparison of the ^1^H NMR spectra of **4** and its C-21 epimer **5** (vide infra) (Table 2). The proximity of the NH nitrogen atom to H-15 in **4** caused a marked downfield shift of H-15 (Δ = 0.30 ppm) (Fig. 4). Hence, the structure of **4** was assigned as shown and named rauvotetraphylline H.

**Table 2:** ^1^H NMR Data for Compounds **4** and **5** (*δ* in ppm, *J* in Hz)

No.	**4**	**5**
3	4.14 (d, 9.7)	4.16 (d, 9.4)
5	3.64 (dd, 6.6, 4.9)	3.63 (dd, 6.1, 4.8)
6*α* 6*β*	1.60 (d, 11.9) 2.79 (dd, 11.9, 4.9)	1.61 (d, 11.9) 2.78 (dd, 11.9, 4.8)
9	7.47 (d, 7.3)	7.45 (d, 7.3)
10	7.22 (dd, 7.6, 7.3)	7.20 (dd, 7.6, 7.3)
11	7.39 (dd, 7.7, 7.6)	7.37 (dd, 7.7, 7.6)
12	7.61 (d, 7.7)	7.60 (d, 7.7)
14*α* 14*β*	1.89 (dd, 14.7, 9.7) 1.52 (dd, 14.7, 4.4)	1.83 (dd, 14.9, 9.4) 1.51 (dd, 14.9, 3.1)
15	2.62 (m)	2.32 (m, overlap)
16	2.34 (dd, 6.6, 5.0)	2.32 (m, overlap)
17	4.99 (s)	4.95 (s)
18	1.29 (d, 6.5)	1.44 (d, 6.4)
19	2.65 (m)	2.76 (m)
20	1.25 (m)	1.20 (m)
21	3.16 (ddd, 11.8, 8.5, 2.7)	3.16 (ddd, 11.2, 8.7, 2.5)
22*α*	2.43 (br. d, 12.9)	2.52 (br. d, 13.4)
22*β*	2.12 (dd, 12.9, 11.8)	1.92 (dd, 13.4, 11.2)
24*α*	2.28 (br. d, 14.4)	2.31 (br. d, 13.5)
24*β*	2.25 (d, 14.4)	2.19 (d, 13.5)
26	1.06 (s)	1.08 (s)
27	1.27 (s)	1.28 (s)
OAc	2.17 (s)	2.16 (s)

Measured in CDCl_3_ (7.26 ppm)

Compound **5**, obtained as amorphous powder, had the same molecular formula as **4**, possessing a quasi-molecular ion peak at *m*/*z* 448.2602 (calcd for C_27_H_34_N_3_O_3_, 448.2600). The ^1^H and ^13^C NMR spectra of **5** (Tables 2 and 3) were almost identical to those of **4** except for the upfield shift of H-15 (Δ = −0.30 ppm) and downfield shifts of Me-18 (Δ = 0.15 ppm) and H-19 (Δ = 0.11 ppm) in the ^1^H NMR spectrum. This can be rationalized in terms of paramagnetic deshielding experienced by Me-18 and H-19 in **5** and H-15 in **4** (Fig. 4), revealing the S* configuration of C-21. This was further supported by significant ROESY correlation (Fig. 4) of H-22*α*↔H-15 and no correlation of H-22*α*↔H-19 or H-22*β*↔Me-18. Therefore, the structure of **5** was elucidated as shown and named 21-*epi*-rauvotetraphylline H.

**Table 3:** ^13^C NMR Data for Compounds **1**–**5** (*δ* in ppm)

No.	**1** ^a^	**2** ^a^	**3** ^b^	**4** ^b^	**5** ^b^
2	139.7 (s)	139.0 (s)	138.3 (s)	183.2 (s)	183.2 (s)
3	44.6 (d)	44.8 (d)	50.0 (d)	56.8 (d)	56.8 (d)
5	53.4 (d)	55.4 (d)	54.8 (d)	50.7 (d)	50.8 (d)
6	28.3 (t)	28.9 (t)	27.6 (t)	37.6 (t)	37.6 (t)
7	104.0 (s)	104.5 (s)	103.9 (s)	64.8 (s)	64.8 (s)
8	128.9 (s)	128.9 (s)	127.5 (s)	136.3 (s)	136.3 (s)
9	118.5 (d)	118.7 (d)	117.9 (d)	123.8 (d)	123.8 (d)
10	119.7 (d)	119.7 (d)	119.4 (d)	125.4 (d)	125.4 (d)
11	121.9 (d)	122.0 (d)	121.4 (d)	128.6 (d)	128.6 (d)
12	111.9 (d)	112.0 (d)	110.9 (d)	120.9 (d)	120.9 (d)
13	138.2 (s)	138.3 (s)	136.3 (s)	156.5 (s)	156.5 (s)
14	34.7 (t)	34.8 (t)	34.5 (t)	22.0 (t)	22.2 (t)
15	29.2 (d)	29.3 (d)	27.4 (d)	27.2 (d)	27.0 (d)
16	48.5 (d)	48.2 (d)	48.1 (d)	49.2 (d)	49.4 (d)
17	56.5 (d)	57.6 (d)	54.8 (d)	78.3 (d)	78.2 (d)
18	14.3 (q)	13.8 (q)	13.6 (q)	20.4 (q)	21.3 (q)
19	125.2 (d)	124.9 (d)	116.4 (d)	53.1 (d)	55.7 (d)
20	137.6 (s)	137.4 (s)	135.8 (s)	49.4 (d)	49.4 (d)
21	91.7 (d)	91.7 (d)	56.0 (t)	51.8 (d)	53.7 (d)
22	47.4 (t)	48.2 (t)	47.2 (t)	47.8 (t)	48.0 (t)
23	213.2 (s)	212.9 (s)	210.4 (s)	209.6 (s)	209.4 (s)
24	55.7 (t)	55.6 (t)	55.4 (t)	54.6 (t)	55.1 (t)
25	55.5 (s)	55.6 (s)	54.5 (s)	54.0 (s)	54.5 (s)
26	25.6 (q)	25.4 (q)	25.2 (q)	25.4 (q)	25.4 (q)
27	31.9 (q)	31.8 (q)	32.2 (q)	32.1 (q)	32.2 (q)
1′	103.2 (d)	103.3 (d)			
2′	75.4 (d)	75.3 (d)			
3′	78.0 (d)	78.0 (d)			
4′	71.4 (d)	71.5 (d)			
5′	77.9 (d)	78.0 (d)			
6′	62.7 (t)	62.7 (t)			
CH_3_COO				21.1 (q)	21.1 (q)
CH_3_COO				170.1 (s)	170.0 (s)

^a^Measured in methanol-*d*_4_ (49.0 ppm)

^b^Measured in CDCl_3_ (77.0 ppm)

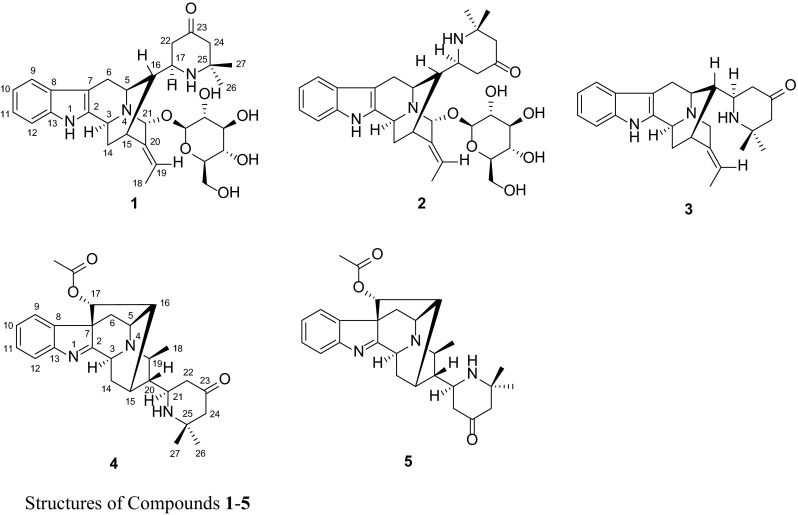

The contribution of artifacts on structural diversity of alkaloids from *Rauvolfia* species is not ignorable as acidic or basic conditions are often used during isolation process, in spite that many artifacts from this genus are generally presented in literatures as naturally occurring compounds [9]. Considering that the presence of aldehyde group at C-16/C-20 is common for sarpagine/perakine type alkaloids [8, 10, 11], it’s plausible to deduce that, like triacetonamine [12], a common artifact of plant extractions, the 2,2-dimethyl-4-oxopiperidine moiety might also be an artifact produced by reaction of aldehyde group with acetone/ammonia since the latter were used as eluents during the isolation procedures. These artifacts represent a unique type of sarpagine/perakine series bearing an unusual piperidine unit brought about by using common eluents.

All of the isolated compounds were evaluated for their in vitro growth inhibitory effects against five human tumor cell lines (HL-60, SMMC-7721, A-549, MCF-7 and SW-480) with cisplatin and taxol serving as positive controls by the MTT method [13]. Regrettably, all tested compounds were inactive (IC_50_ values > 40 μM).

## Experimental Section

### General Experimental Procedures

Optical rotations were measured on a Jasco P-1020 automatic digital polarimeter. UV data were obtained from online HPLC analysis. IR spectra (KBr) were obtained on a Bruker Tensor-27 infrared spectrophotometer. NMR spectra were acquired with a Bruker DRX-500 or Bruker Avance III 600 instrument (Bruker BioSpin GmbH, Rheinstetten, Germany) with deuterated solvent signals used as internal standards. ESI-MS (including HR-ESI-MS) were measured on API QSTAR Pulsar i mass spectrometers. Silica gel (200–300 mesh, Qingdao Marine Chemical Inc., China) and Sephadex LH-20 (Amersham Biosciences, Sweden) were used for column chromatography. Medium pressure liquid chromatography (MPLC) was performed on a Büchi Sepacore System equipping with pump manager C-615, pump modules C-605, and fraction collector C-660 (Büchi Labortechnik AG, Switzerland), and columns packed with Chromatorex C-18 (40–75 μm, Fuji Silysia Chemical Ltd., Japan). Fractions were monitored by TLC (Qingdao Marine Chemical Inc., China) in combination with reversed-phase HPLC (Agilent 1200, Extend-C18 column, 5 μm, 4.6 × 150 mm).

### Plant Material

The aerial parts of *Rauvolfia tetraphylla* were collected in Xiaomenglun of Yunnan Province, China, in June 2010 and identified by Mr. Yu Chen of Kunming Institute of Botany, Chinese Academy of Sciences. The voucher specimen (No. BBP0234020RT) was deposited at BioBioPha Co., Ltd.

### Extraction and Isolation

The air-dried and powdered aerial parts of *R. tetraphylla* (7.5 kg) were extracted three times with EtOH-H_2_O (95:5, v/v; 3 × 20 L, each 5 days) at room temperature, and the solvent was removed under reduced pressure to give crude extract (ca. 400 g), which was then fractionated by silica gel column chromatography (CC) eluted with a gradient solvent system (containing 0.2 % ammonia) of petroleum ether-acetone and then MeOH to yield seven fractions A–G. Fraction D, eluted by acetone, was separated on silica gel CC (CHCl_3_–MeOH–ammonia, 100:1:0.5 → 0:100:0.5) to give three subfractions D1–D3. Fraction D1 was purified further by silica gel CC (CHCl_3_–MeOH–ammonia, 50:1:0.1) and then prep. TLC (CHCl_3_–MeOH–ammonia, 10:1:0.1) to afford **4** (9 mg) and **5** (6 mg). Fraction D2 was separated by silica gel CC (CHCl_3_–MeOH–ammonia, 40:1:0.1) and then prep. TLC (CHCl_3_–MeOH–ammonia, 9:1:0.1) to afford **3** (10 mg). Fraction G, eluted by MeOH, was separated further by silica gel CC (CHCl_3_–MeOH–ammonia, 10:1:0.1 → 0:10:0.1), repeated MPLC (40 → 45 % MeOH in H_2_O), and then Sephadex LH-20 (MeOH) to afford **1** (72 mg) and **2** (26 mg).

### Rauvotetraphylline F (**1**)

White amorphous powder; [α]_D_^15^ +10.1 (*c* 0.19, CHCl_3_); UV (MeOH) *λ*_max_: 225, 281, 290 (sh) nm; IR (KBr) *ν*_max_ 3404, 2961, 2921, 1700, 1470, 1453, 1384, 1338, 1320, 1302, 1076, 1031, 745 cm^−1^; ^1^H and ^13^C NMR data see Tables 1 and 3; ESI-MS (pos.): *m/z* 568 [M + H]^+^; HR-ESI-MS (pos.): *m/z* 568.3025 (calcd for C_31_H_42_N_3_O_7_, 568.3022).

### 17-*epi*-Rauvotetraphylline F (**2**)

White amorphous powder; [α]_D_^16^ +14.9 (*c* 0.20, CHCl_3_); UV (MeOH) *λ*_max_: 225, 281, 290 (sh) nm; IR (KBr) *ν*_max_ 3396, 2962, 2923, 1699, 1626, 1471, 1451, 1384, 1337, 1300, 1075, 1030, 746 cm^−1^; ^1^H and ^13^C NMR data see Tables 1 and 3; ESI-MS (pos.): *m/z* 568 [M + H]^+^; HR-ESI-MS (pos.): *m/z* 568.3031 (calcd for C_31_H_42_N_3_O_7_, 568.3022).

### Rauvotetraphylline G (**3**)

White amorphous powder; [α]_D_^14^ −22.4 (*c* 0.19, CHCl_3_); UV (MeOH) *λ*_max_: 225, 280, 290 (sh) nm; IR (KBr) *ν*_max_ 3421, 3143, 3057, 2961, 2925, 2855, 1705, 1626, 1473, 1301, 1240, 1169, 741 cm^−1^; ^1^H and ^13^C NMR data see Tables 1 and 3; ESI-MS (pos.): *m/z* 390 [M + H]^+^; HR-ESI-MS (pos.): *m/z* 390.2538 (calcd for C_25_H_32_N_3_O, 390.2545).

### Rauvotetraphylline H (**4**)

White amorphous powder; [α]_D_^14^ +9.9 (*c* 0.20, CHCl_3_); UV (MeOH) *λ*_max_: 220, 262 nm; IR (KBr) *ν*_max_ 3433, 2965, 2934, 1741, 1707, 1592, 1453, 1380, 1364, 1295, 1033, 773, 753 cm^−1^; ^1^H and ^13^C NMR data see Tables 2 and 3; ESI-MS (pos.): *m/z* 448 [M + H]^+^; HR-ESI-MS (pos.): *m/z* 448.2613 (calcd for C_27_H_34_N_3_O_3_, 448.2600).

### 17-*epi*-Rauvotetraphylline H (**5**)

White amorphous powder; [α]_D_^15^ +47.3 (*c* 0.20, CHCl_3_); UV (MeOH) *λ*_max_: 220, 263 nm; IR (KBr) *ν*_max_ 3433, 2965, 2936, 1742, 1707, 1592, 1453, 1376, 1268, 1230, 1177, 1032, 774, 753 cm^−1^; ^1^H and ^13^C NMR data see Tables 2 and 3; ESI-MS (pos.): *m/z* 448 [M + H]^+^; HR-ESI-MS (pos.): *m/z* 448.2602 (calcd for C_27_H_34_N_3_O_3_, 448.2600).

### Cytotoxicity Bioassay

The cytotoxicity assay was performed according to an MTT [3-(4,5-dimethylthiazol-2-yl)-2,5-diphenyltetrazolium bromide] method [14], by use of the following five human cancer cell lines: HL-60, SMMC-7721, A-549, MCF-7, and SW-480. The IC_50_ values were calculated by Reed and Muench’s method [15].

## Electronic supplementary material

Supplementary material 1 (DOCX 2343 kb)
