# Supplementary material for: Hybrid Monoterpenoid Indole Alkaloids Obtained as Artifacts from Rauvolfia tetraphylla
Source: Nat Prod Bioprospect. 2015 Sep 29;5(5):247–53. doi: 10.1007/s13659-015-0074-2 (PMC4607677; doi:10.1007/s13659-015-0074-2)

**Hybrid monoterpenoid indole alkaloids obtained as artifacts from *Rauvolfia tetraphylla***

Yuan Gao ^a,b^ • Dong-Sheng Zhou ^a^ • Ping Hai ^a^ • Yan Li ^c^ • Fei Wang ^a^^,^*

^a^ BioBioPha Co., Ltd., Kunming 650201, People’s Republic of China

^b^ Department of Chemical Engineering, Yibin University, Yibin 644000, People’s Republic of China

^c^ State Key Laboratory of Phytochemistry and Plant Resources in West China, Kunming Institute of Botany, Chinese Academy of Sciences, Kunming 650201, People’s Republic of China

BioBioPha Co., Ltd., Kunming 650201, People’s Republic of China

E-mail: f.wang@mail.biobiopha.com

Structures of Compounds **1**-**5**

**Content list:**

**S1.** ^1^H NMR spectrum (600 MHz, methanol-*d*_6_) of **1**.

**S2.** ^13^C NMR (DEPT) spectrum (150 MHz, methanol-*d*_4_) of **1**.

**S3.** HMBC spectrum (600 MHz, methanol-*d*_4_) of **1**.

**S4.** HSQC spectrum (600 MHz, methanol-*d*_4_) of **1**.

**S5.** ^1^H-^1^H COSY spectrum (600 MHz, methanol-*d*_4_) of **1**.

**S6.** ROESY spectrum (600 MHz, methanol-*d*_4_) of **1**.

**S7.** ^1^H NMR spectrum (600 MHz, methanol-*d*_4_) of **2**.

**S8.** ^13^C NMR (DEPT) spectrum (150 MHz, methanol-*d*_4_) of **2**.

**S9.** HMBC spectrum (600 MHz, methanol-*d*_4_) of **2**.

**S10.** HSQC spectrum (600 MHz, methanol-*d*_4_) of **2**.

**S11.** ROESY spectrum (600 MHz, methanol-*d*_4_) of **2**.

**S12.** ^1^H NMR spectrum (600 MHz, CDCl_3_) of **3**.

**S13.** ^13^C NMR (DEPT) spectrum (150 MHz, CDCl_3_) of **3**.

**S14.** HMBC spectrum (600 MHz, CDCl_3_) of **3**.

**S15.** ROESY spectrum (600 MHz, CDCl_34_) of **3**.

**S16.** ^1^H NMR spectrum (600 MHz, CDCl_3_) of **4**

**S17.** ^13^C NMR (DEPT) spectrum (150 MHz, CDCl_3_) of **4**.

**S18.** HMBC spectrum (600 MHz, CDCl_3_) of **4**.

**S19.** ROESY spectrum (600 MHz, CDCl_3_) of **4**.

**S20.** ^1^H NMR spectrum (500 MHz, CDCl_3_) of **5**.

**S21.** ^13^C NMR (DEPT) spectrum (150 MHz, CDCl_3_) of **5**.

**S22.** HMBC spectrum (600 MHz, CDCl_3_) of **5**.

**S23.** ROESY spectrum (600 MHz, CDCl_3_) of **5**.

**S1.** ^1^H NMR spectrum (600 MHz, methanol-*d*_6_) of **1**.

**
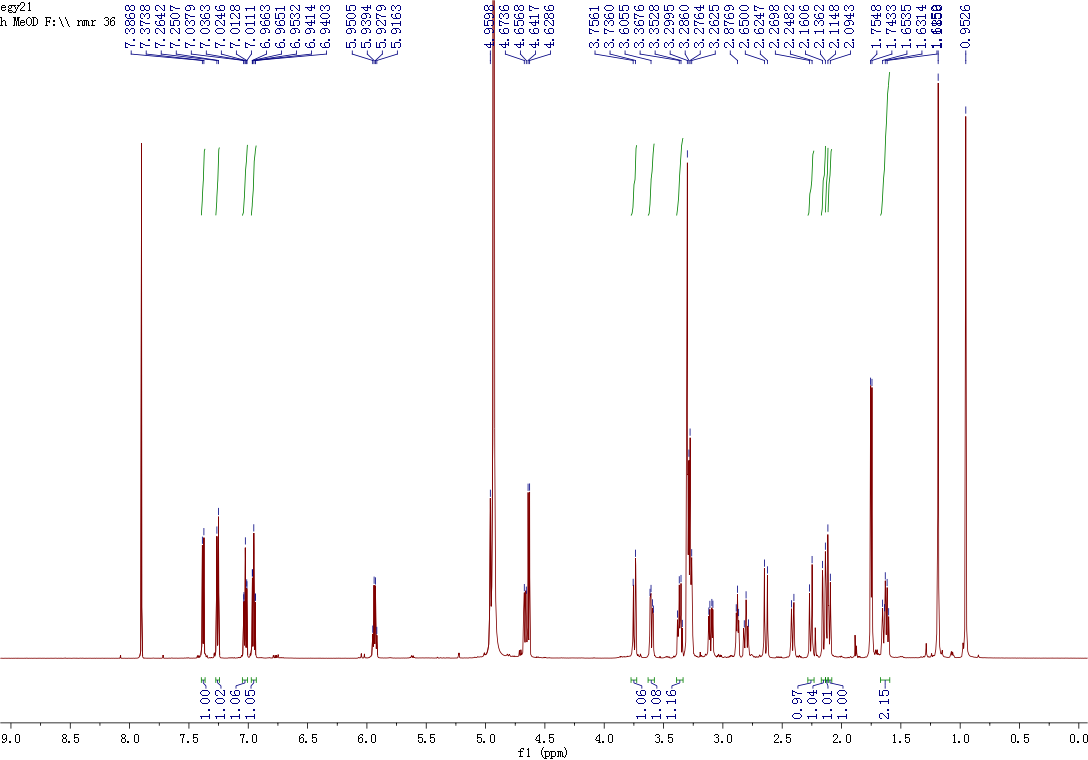
**

**S2.** ^13^C NMR (DEPT) spectrum (150 MHz, methanol-*d*_4_) of **1**.

**
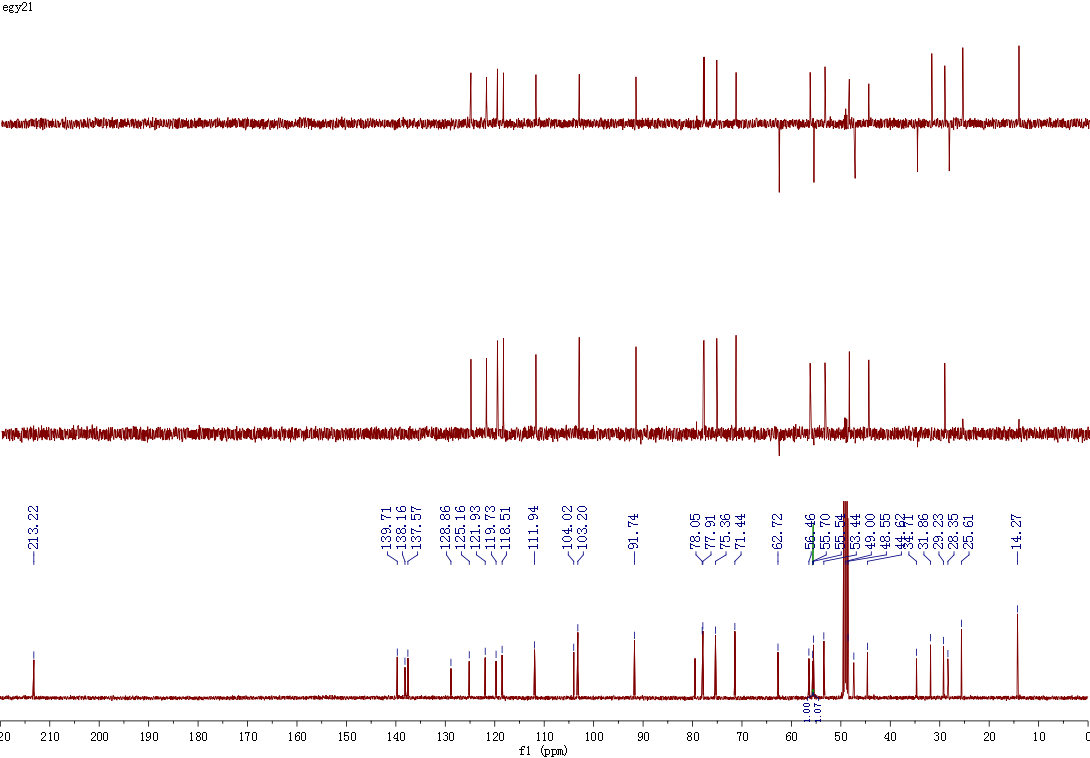
**

**S3.** HMBC spectrum (600 MHz, methanol-*d*_4_) of **1**.


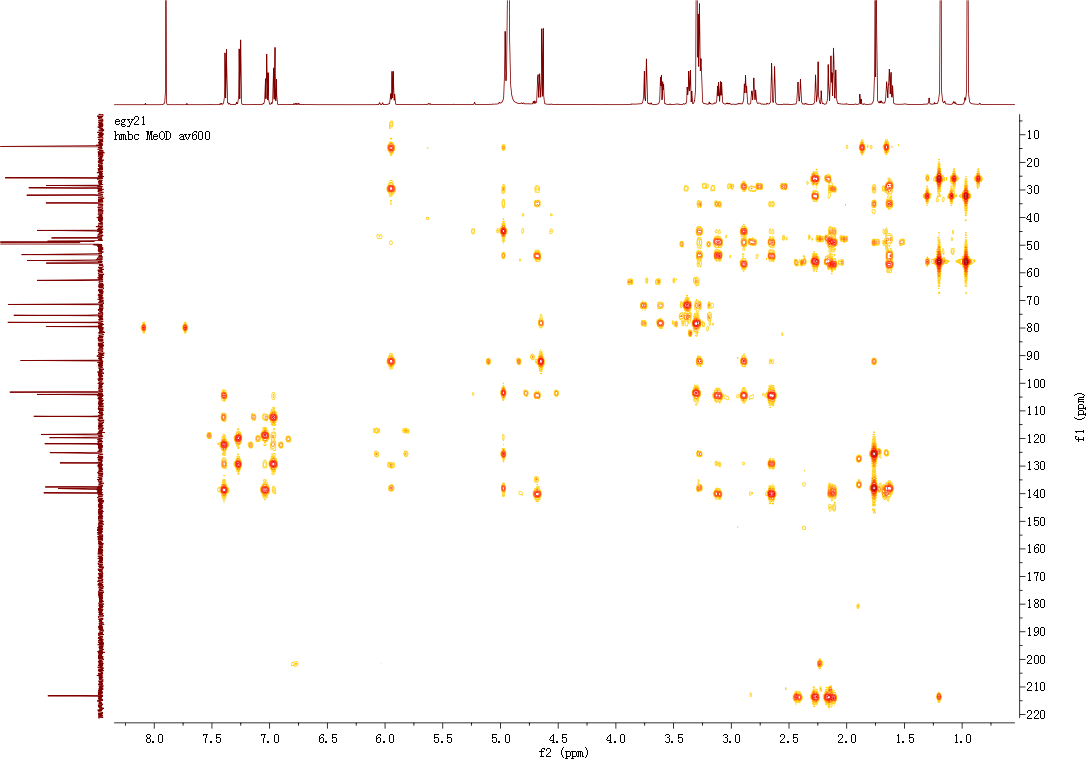

**S4.** HSQC spectrum (600 MHz, methanol-*d*_4_) of **1**.


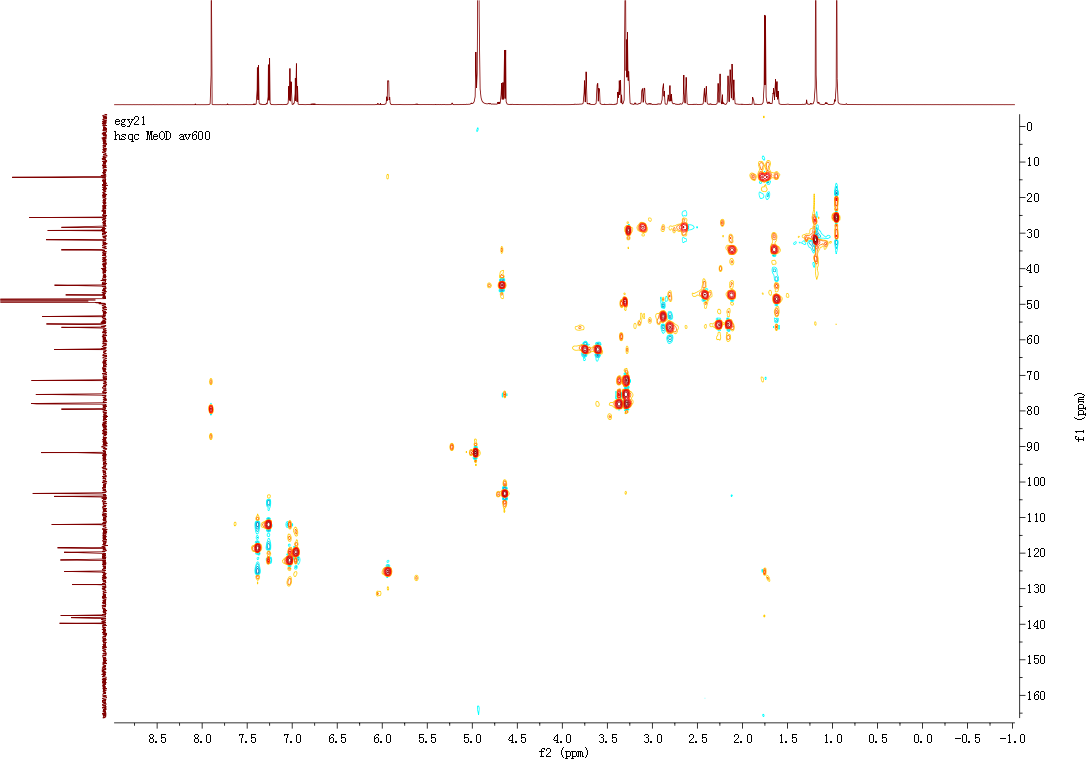

**S5.** ^1^H-^1^H COSY spectrum (600 MHz, methanol-*d*_4_) of **1**.


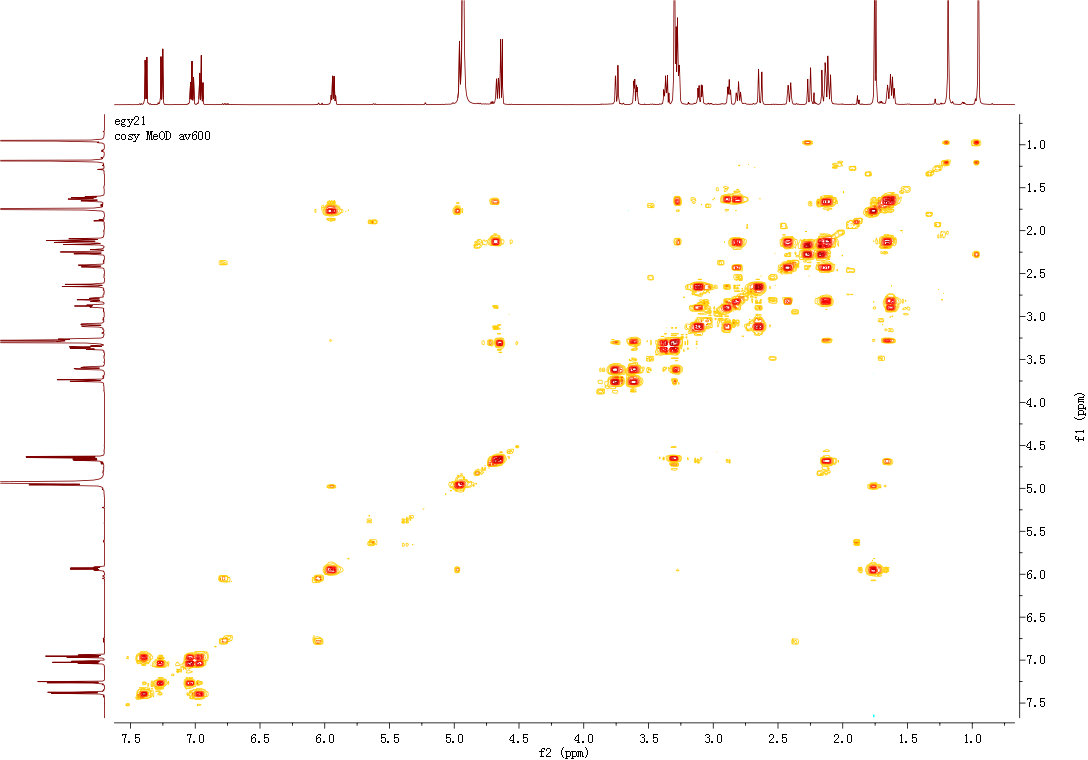

**S6.** ROESY spectrum (600 MHz, methanol-*d*_4_) of **1**.


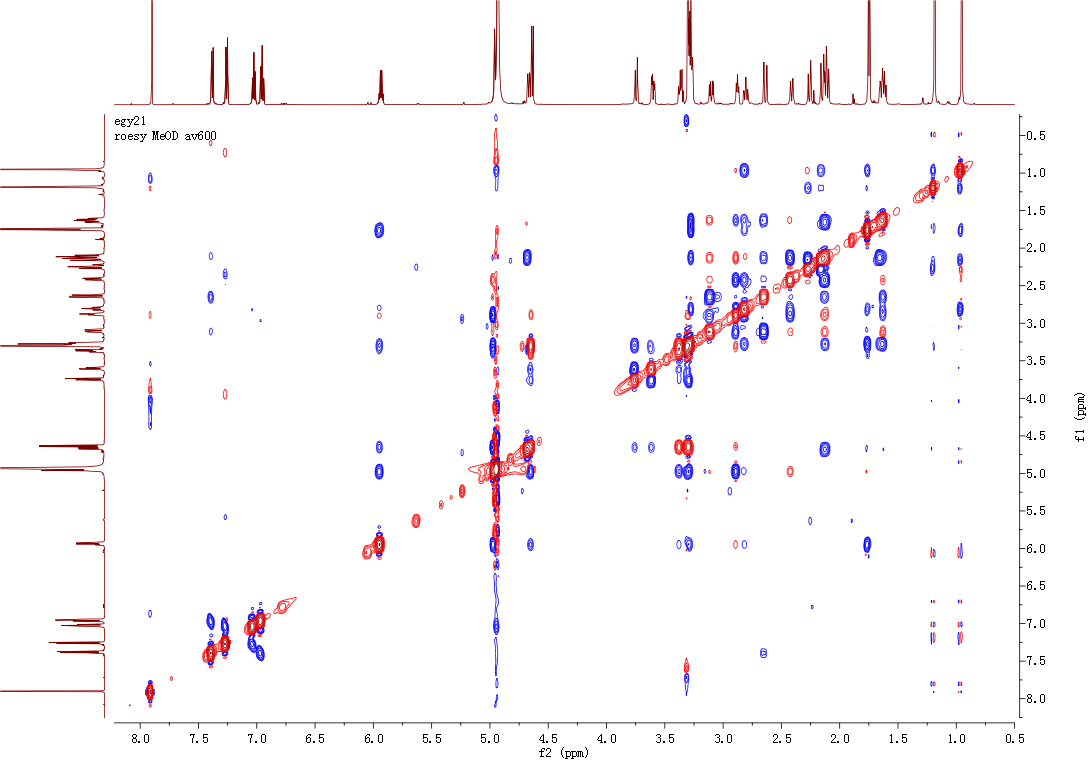

**S7.** ^1^H NMR spectrum (600 MHz, methanol-*d*_4_) of **2**.


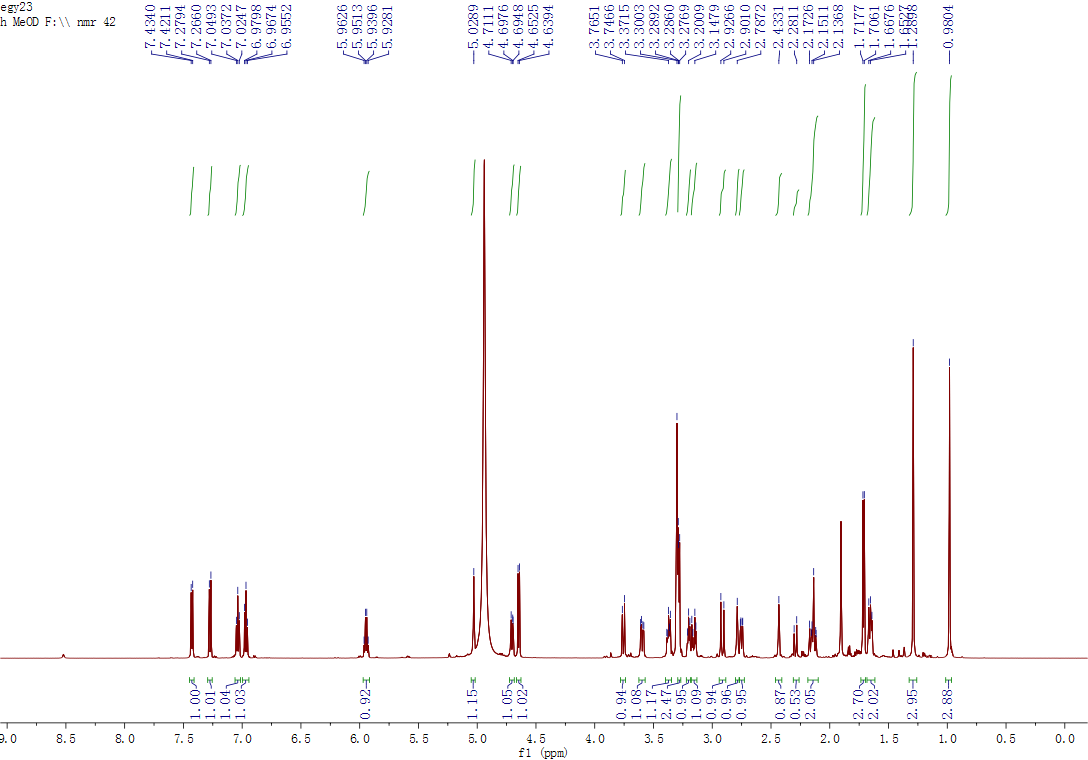

**S8.** ^13^C NMR (DEPT) spectrum (150 MHz, methanol-*d*_4_) of **2**.

**
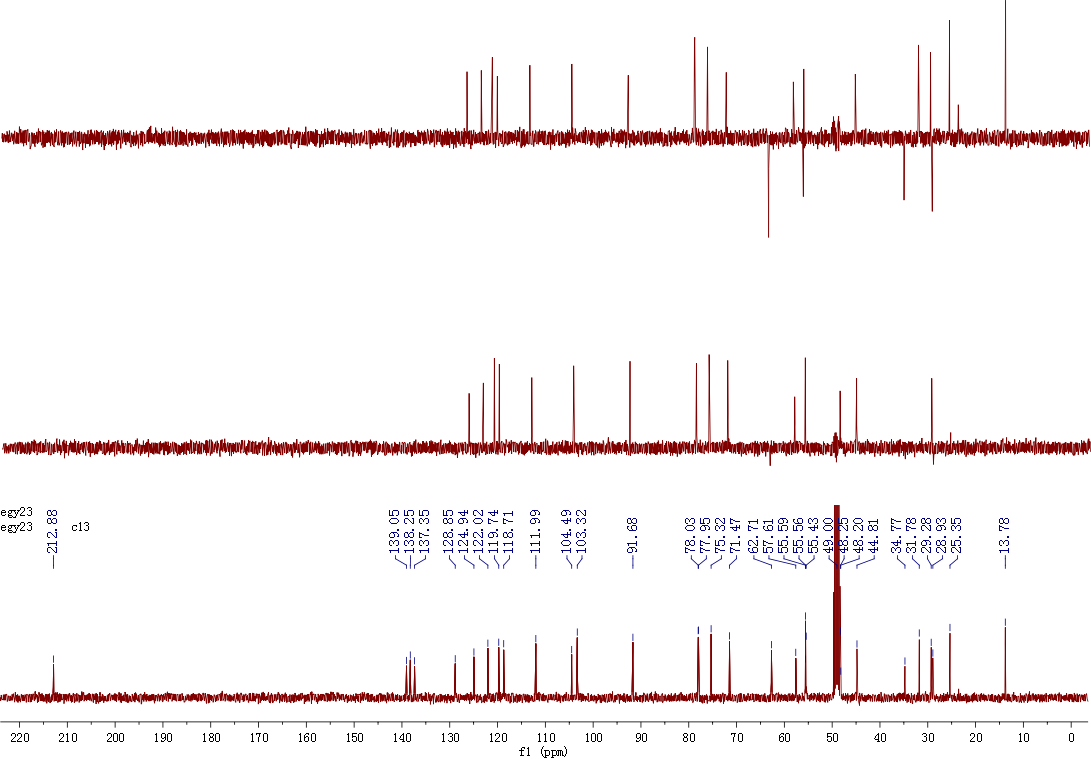
**

**S9.** HMBC spectrum (600 MHz, methanol-*d*_4_) of **2**.


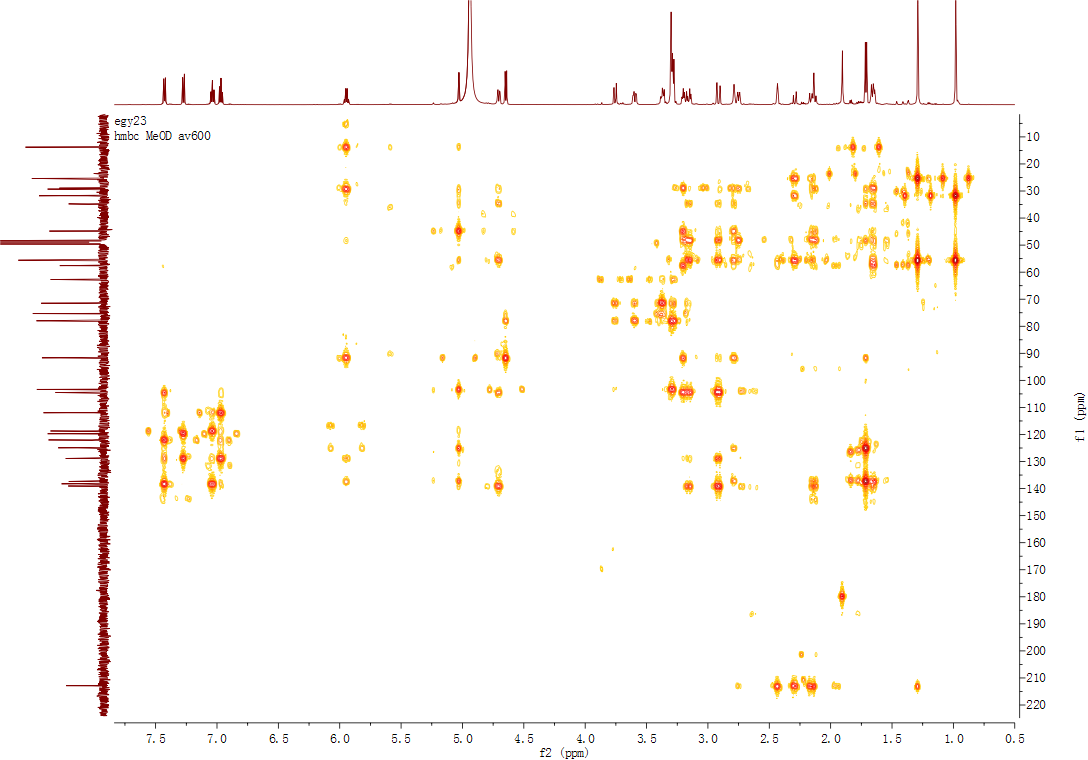

**S10.** HSQC spectrum (600 MHz, methanol-*d*_4_) of **2**.


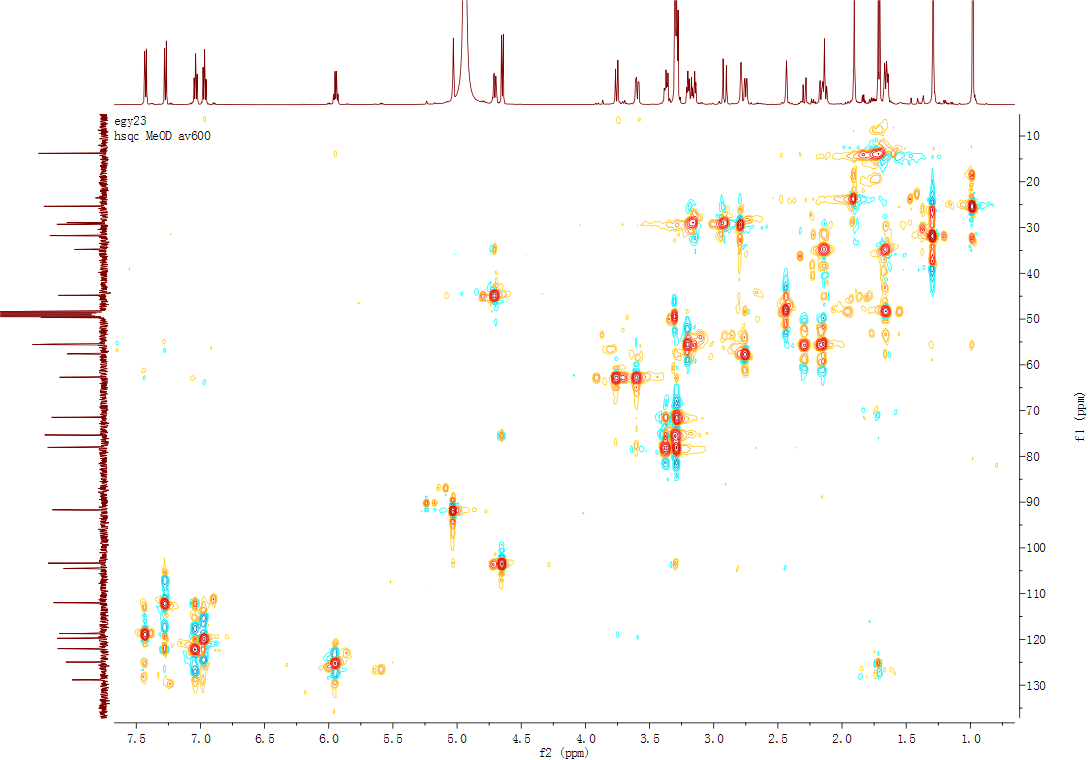

**S11.** ROESY spectrum (600 MHz, methanol-*d*_4_) of **2**.


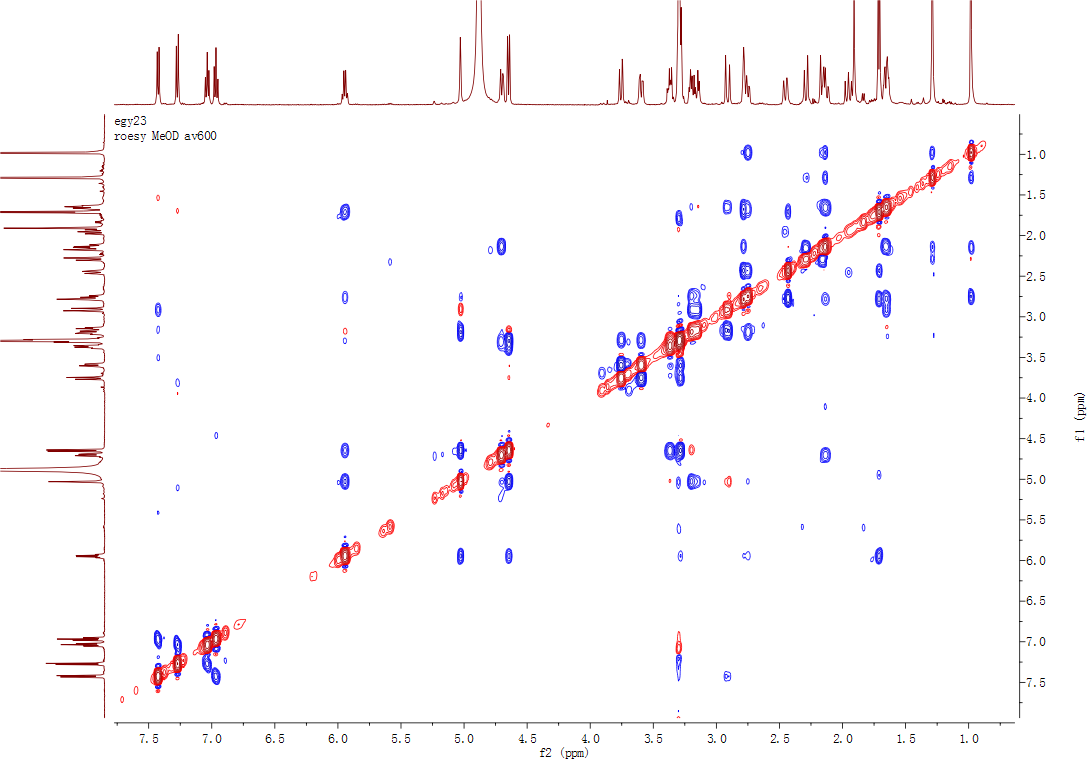

**S12.** ^1^H NMR spectrum (600 MHz, CDCl_3_) of **3**.

**
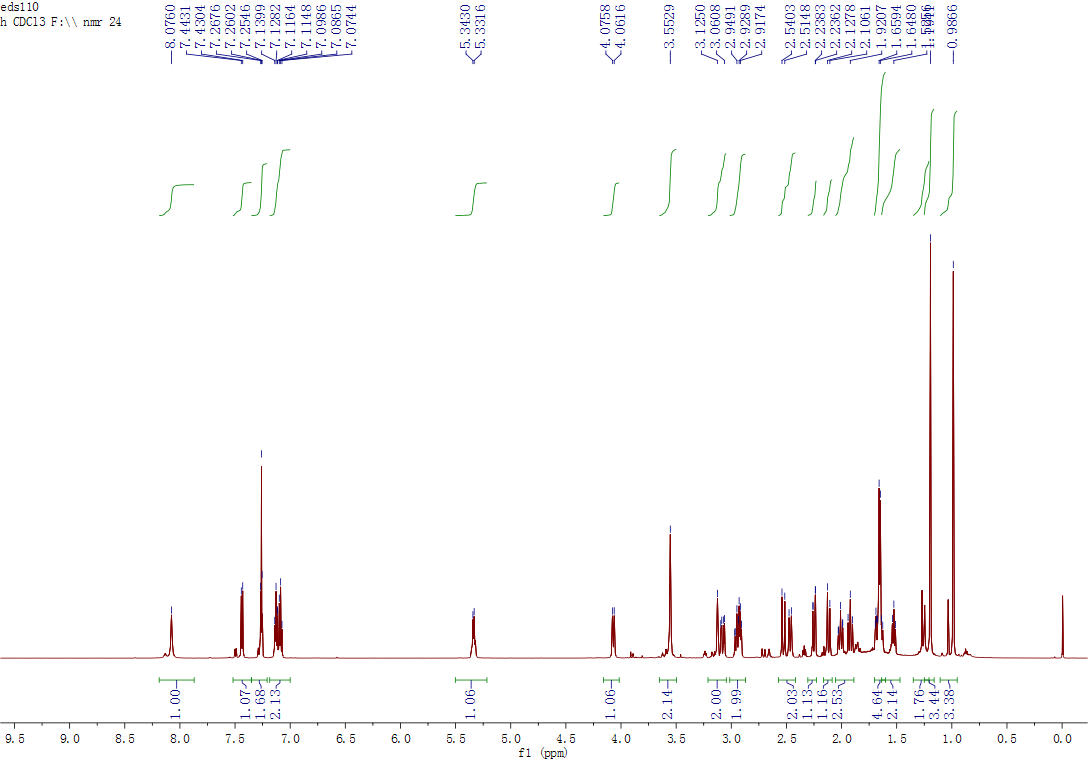
**

**S13.** ^13^C NMR (DEPT) spectrum (150 MHz, CDCl_3_) of **3**.


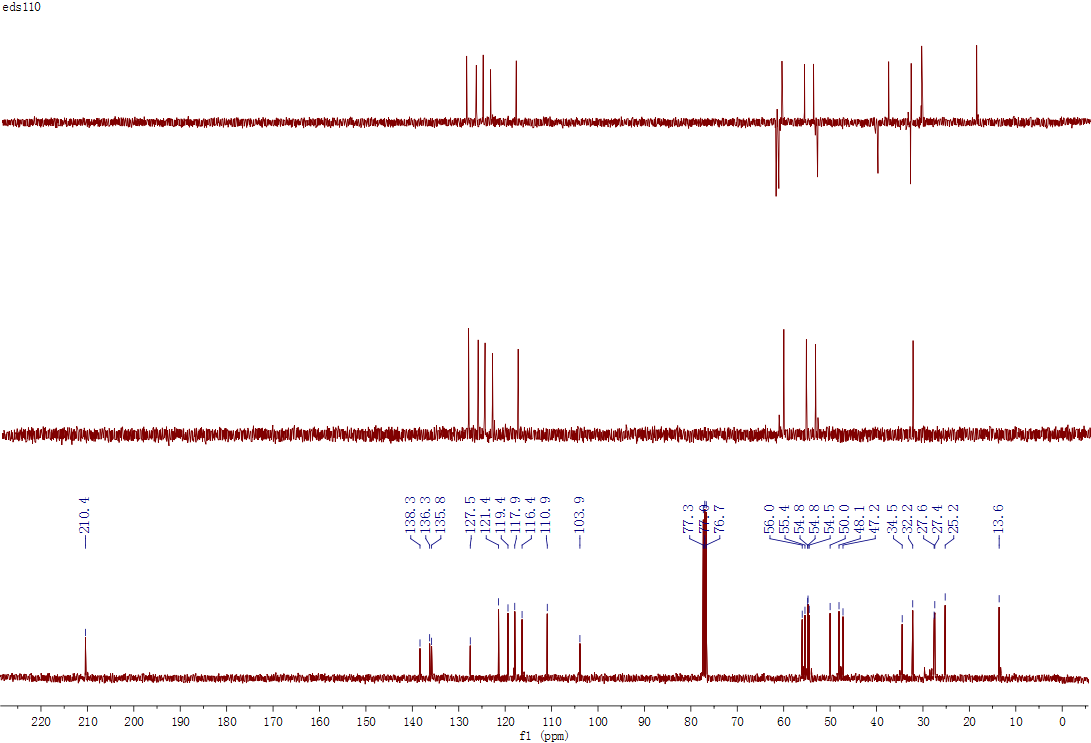

**S14.** HMBC spectrum (600 MHz, CDCl_3_) of **3**.


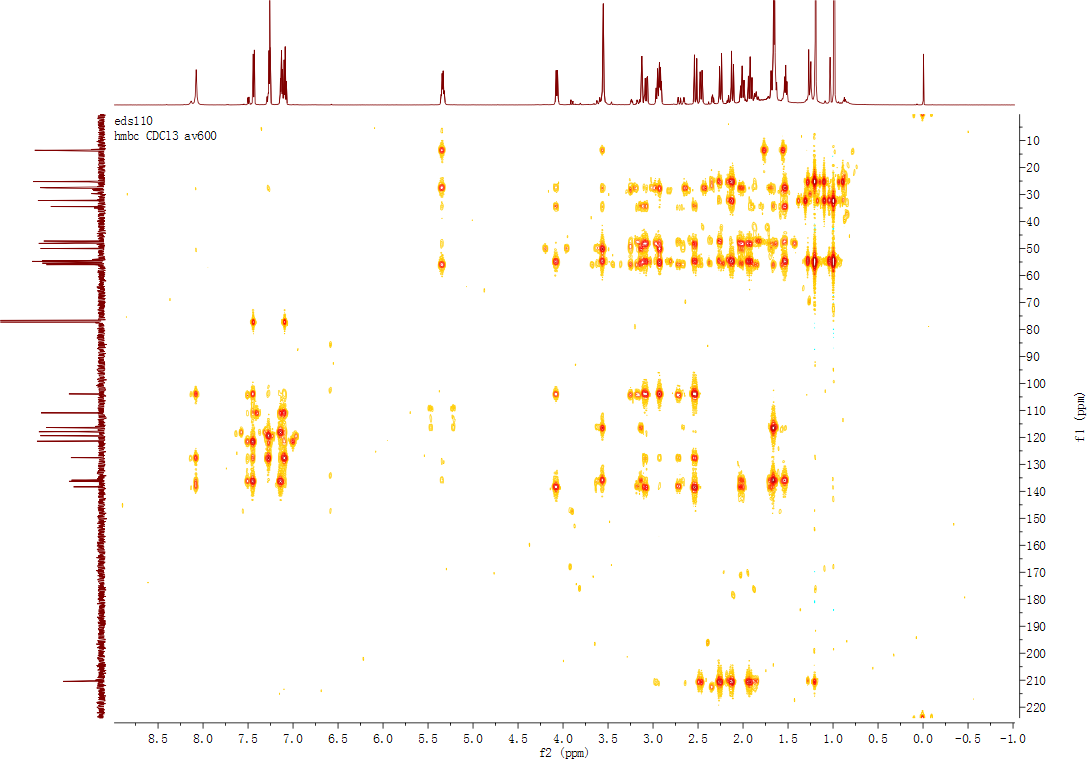

**S15.** ROESY spectrum (600 MHz, CDCl_34_) of **3**.


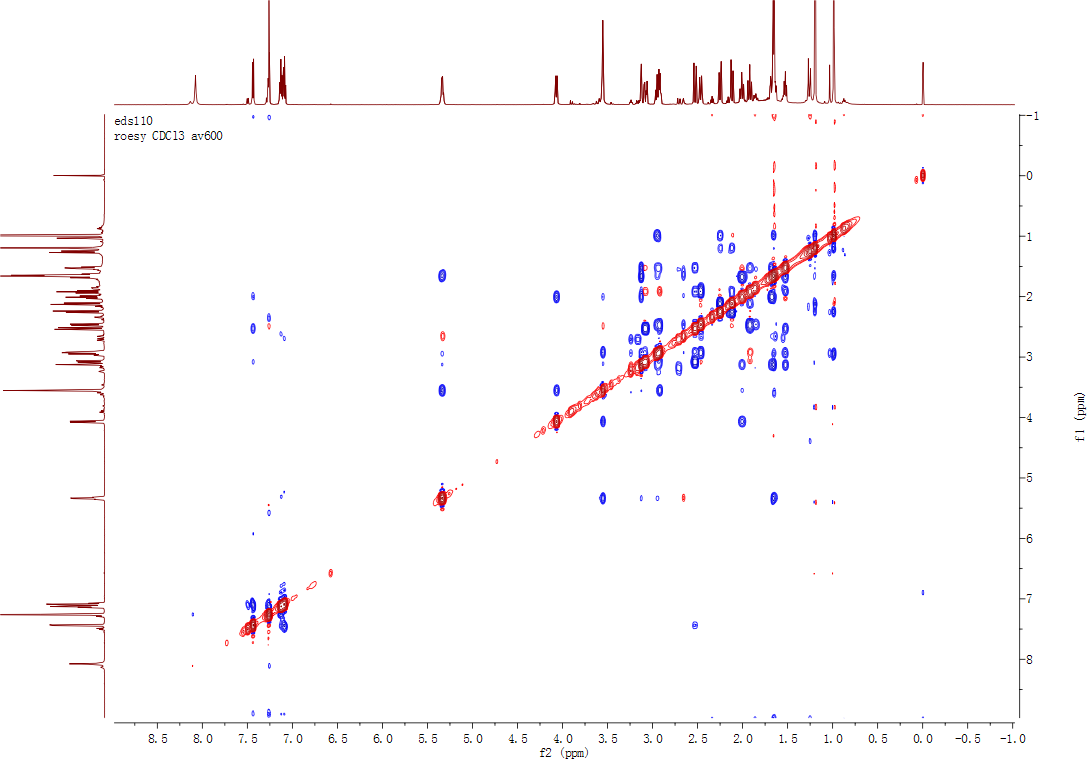

**S16.** ^1^H NMR spectrum (600 MHz, CDCl_3_) of **4**

**
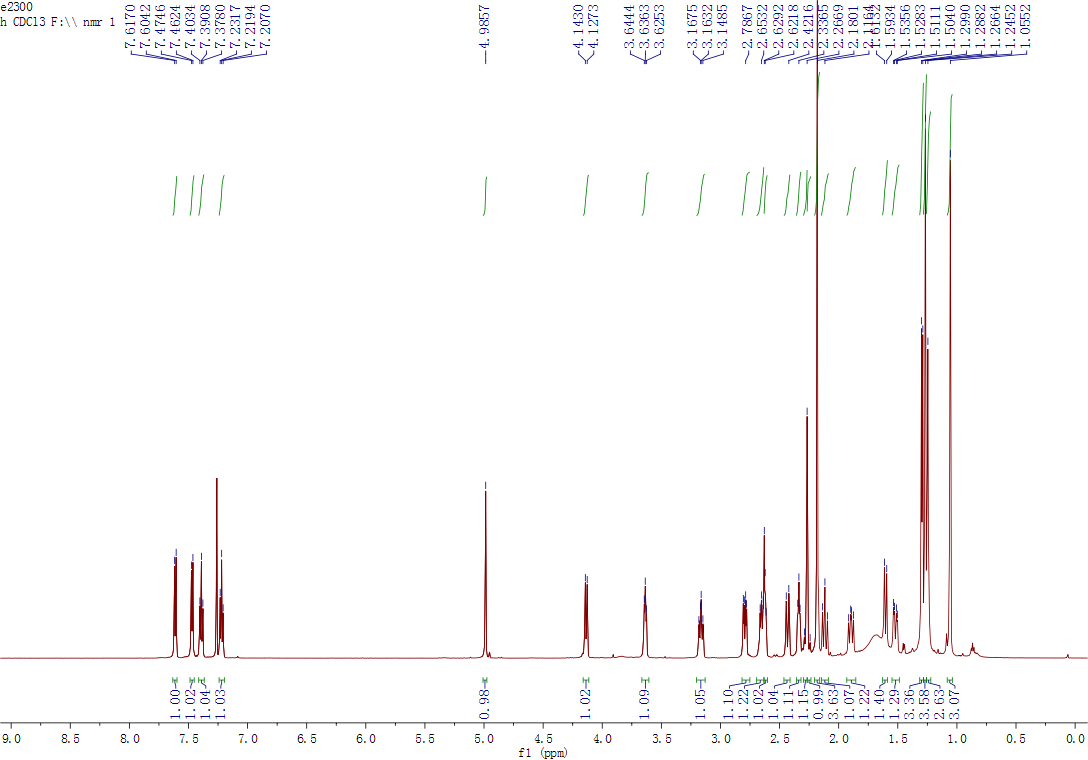
**

**S17.** ^13^C NMR (DEPT) spectrum (150 MHz, CDCl_3_) of **4**.


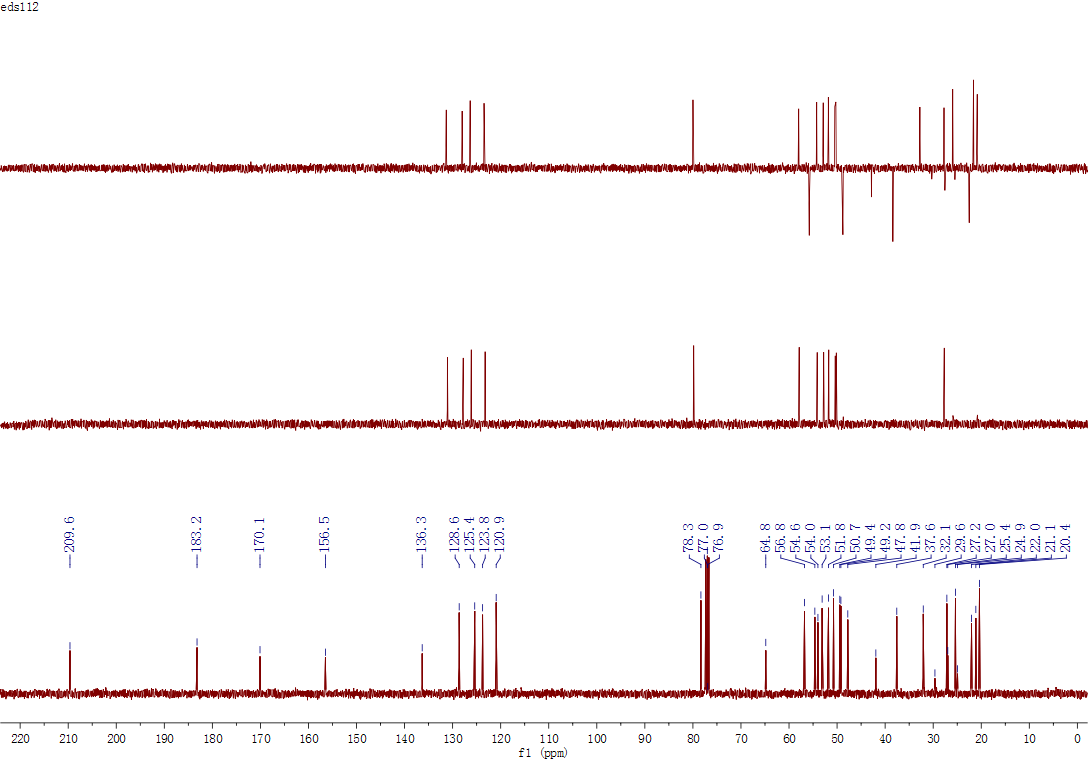

**S18.** HMBC spectrum (600 MHz, CDCl_3_) of **4**.


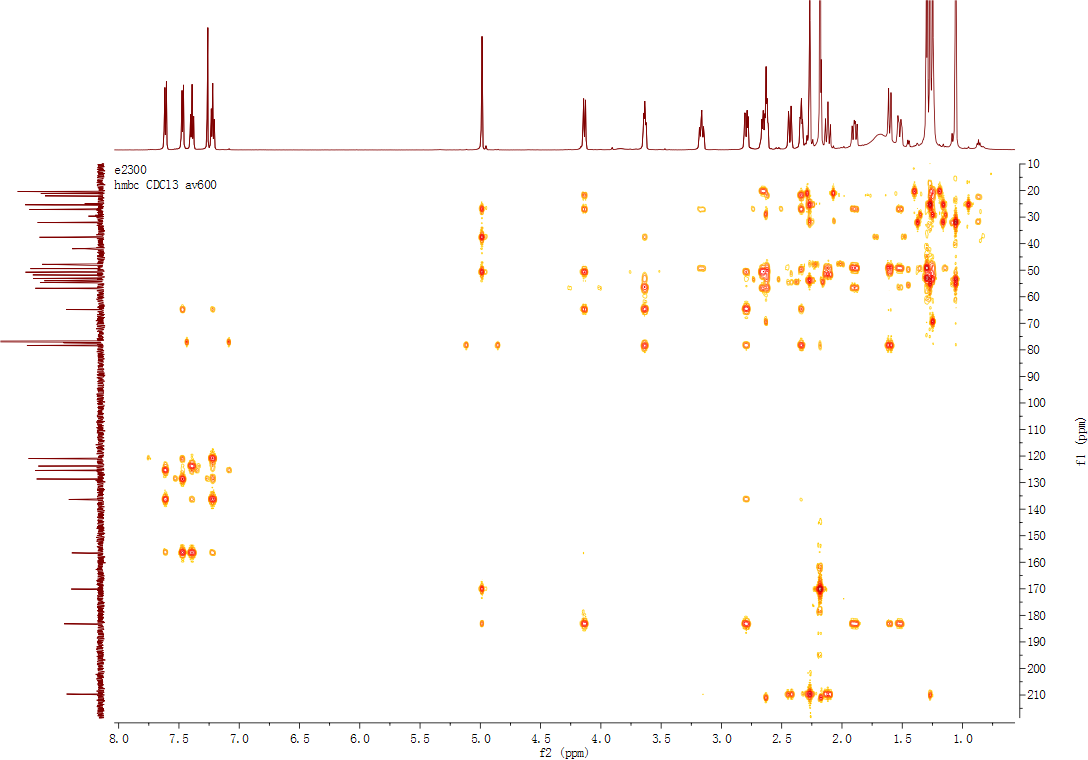

**S19.** ROESY spectrum (600 MHz, CDCl_3_) of **4**.


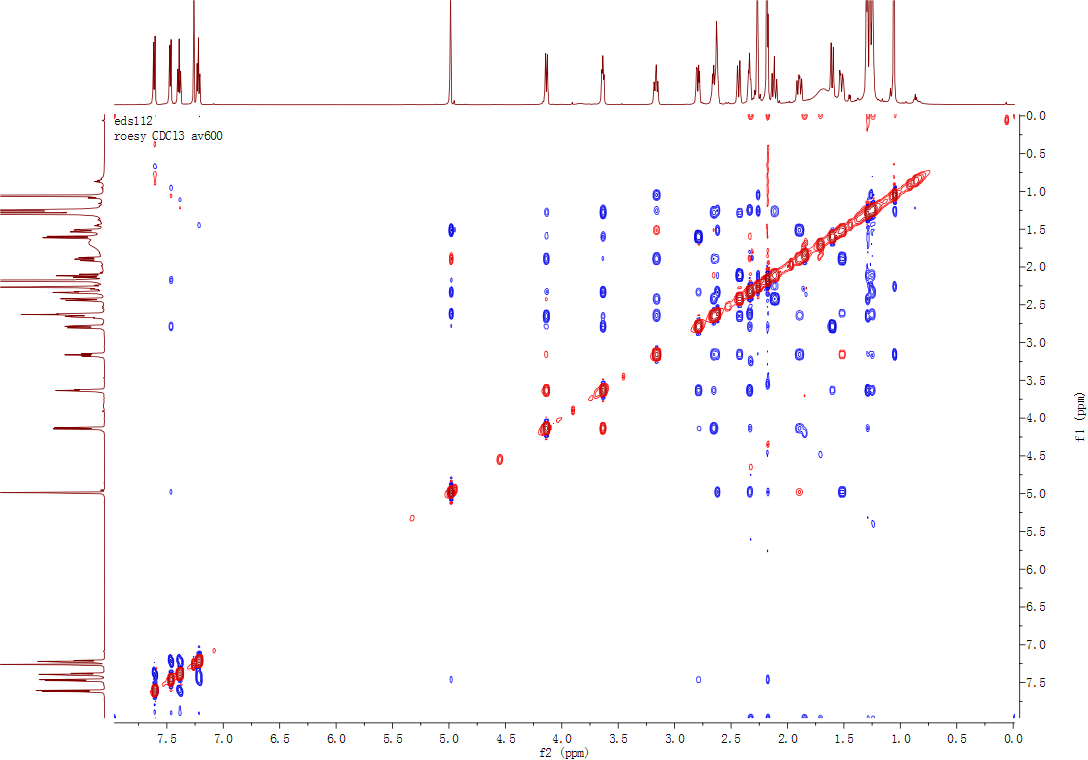

**S20.** ^1^H NMR spectrum (500 MHz, CDCl_3_) of **5**

**
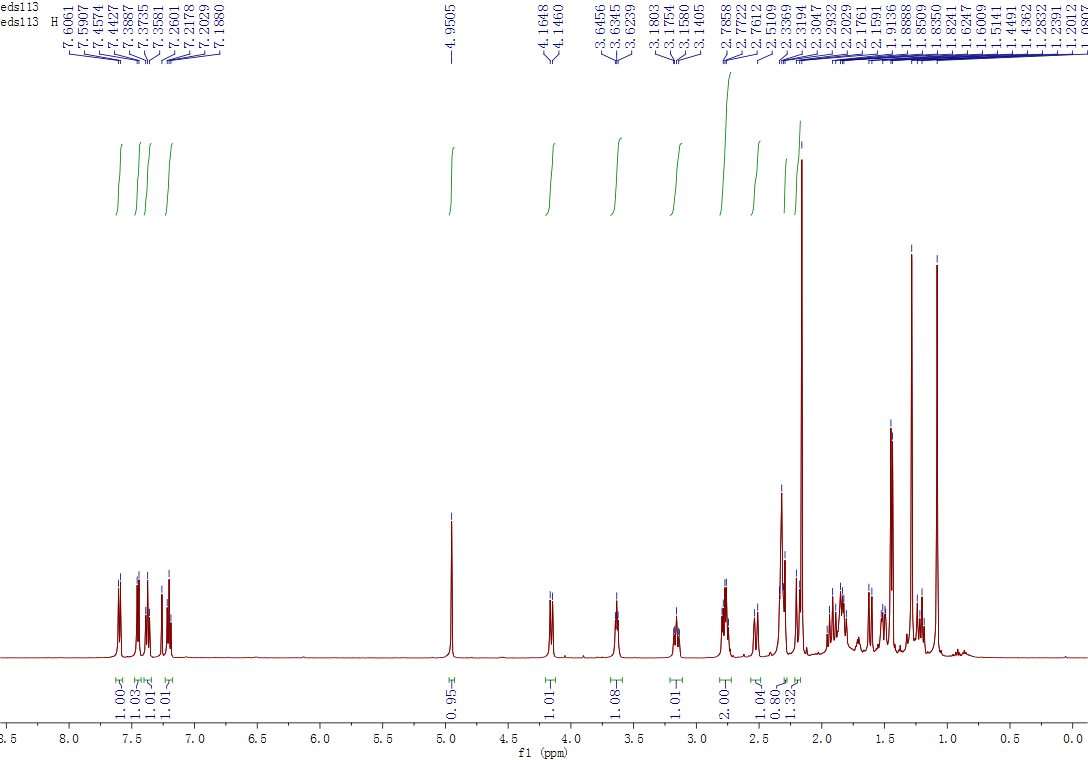
**

**S21.** ^13^C NMR (DEPT) spectrum (150 MHz, CDCl_3_) of **5**.


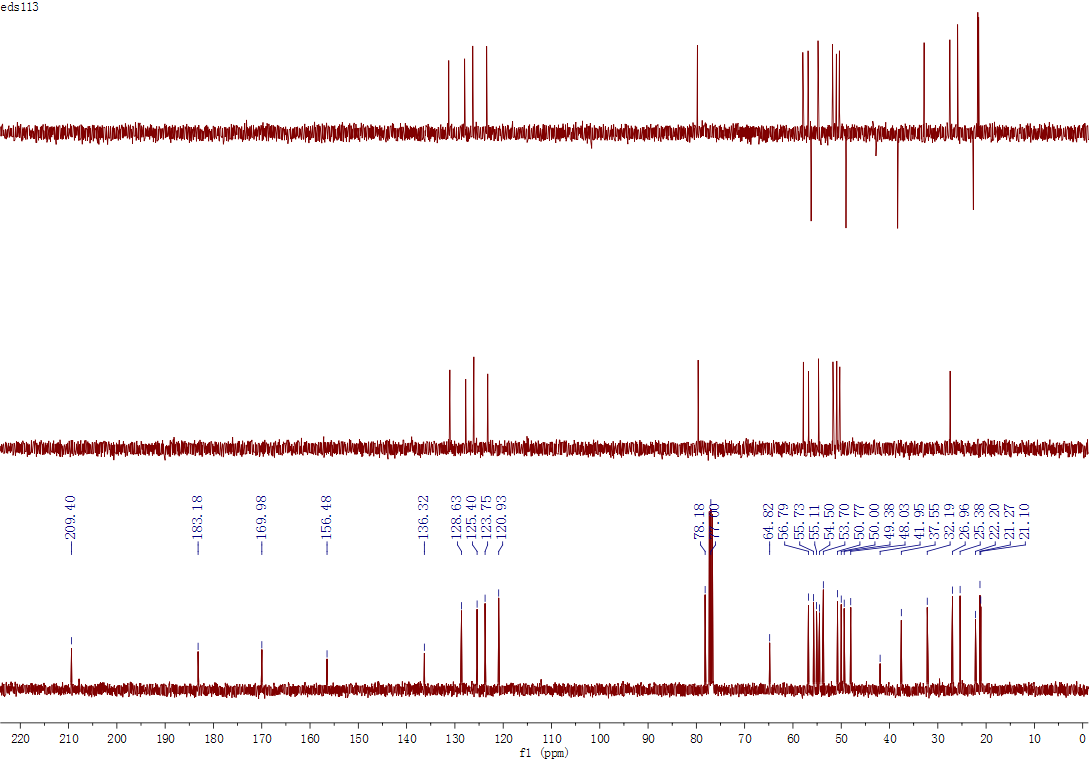

**S22.** HMBC spectrum (600 MHz, CDCl_3_) of **5**.


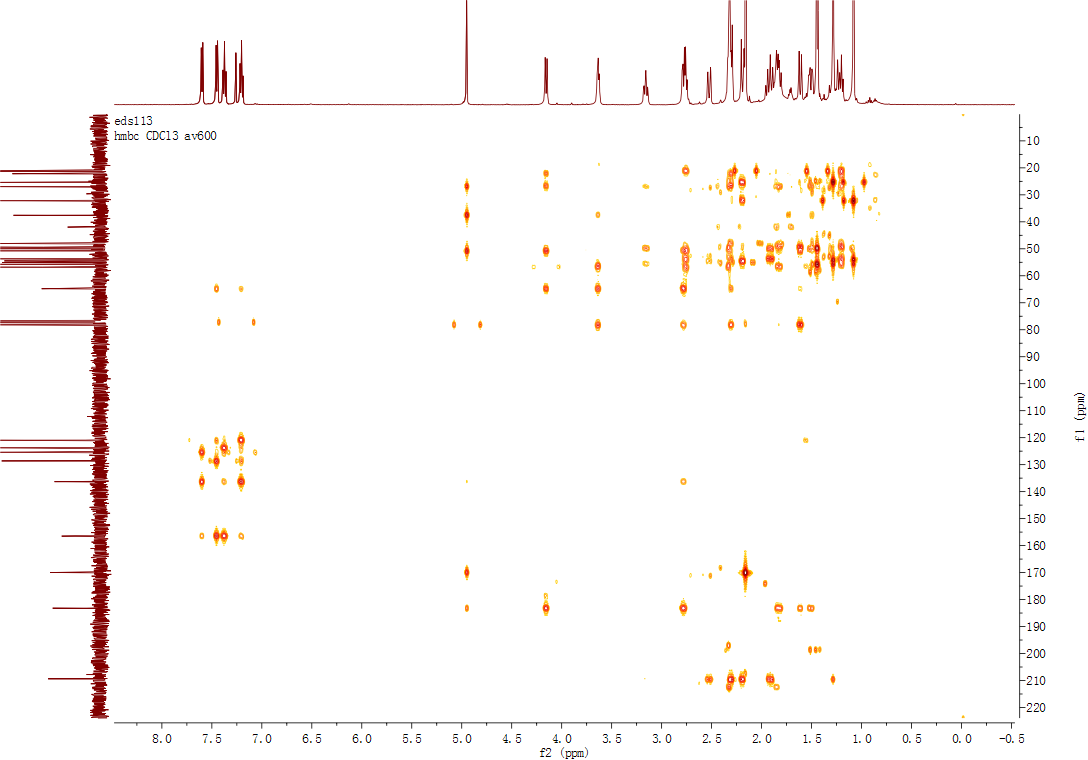

**S23.** ROESY spectrum (600 MHz, CDCl_3_) of **5**.


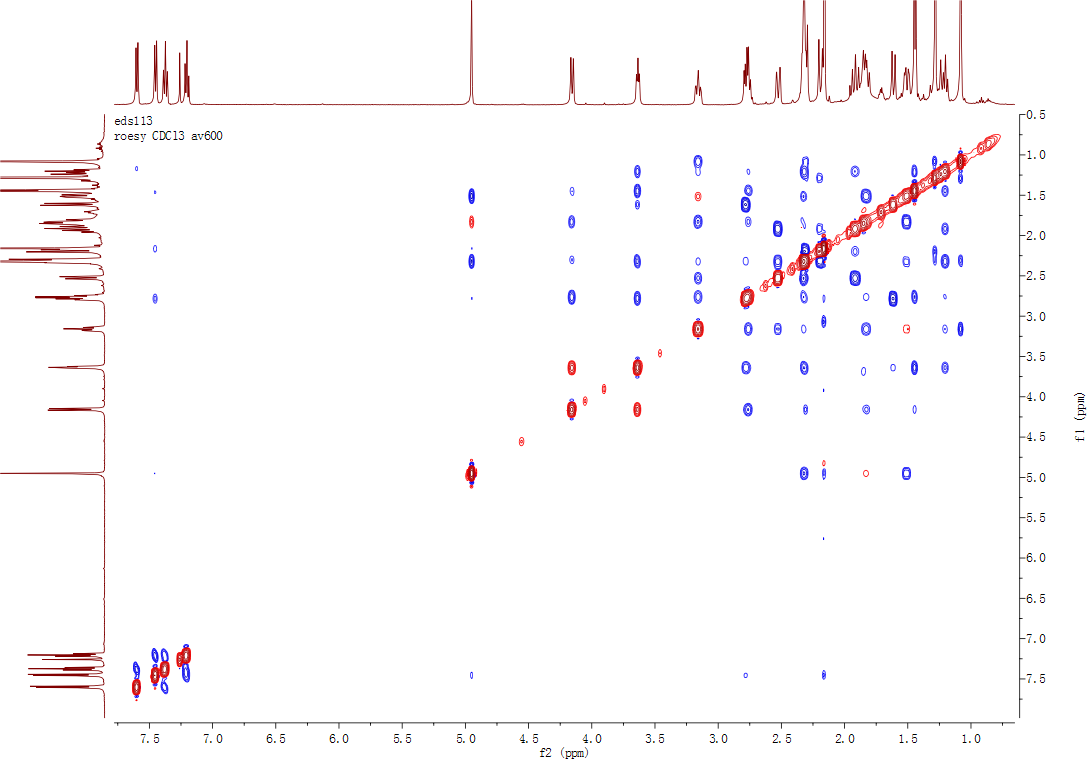

Supplement: Supplementary file 1 — Supplementary material 1 (DOCX 2343 kb) [file 13659_2015_74_MOESM1_ESM.docx]
